# Protective Effects of Genistein against Mono-(2-ethylhexyl) Phthalate-Induced Oxidative Damage in Prepubertal Sertoli Cells

**DOI:** 10.1155/2017/2032697

**Published:** 2017-11-13

**Authors:** Liandong Zhang, Ming Gao, Tongdian Zhang, Tie Chong, Ziming Wang, Xiaoqiang Zhai, Zhizhong Wu, Hecheng Li

**Affiliations:** ^1^Department of Urology, The Second Affiliated Hospital, Xi'an Jiaotong University, Xi'an, Shaanxi 710004, China; ^2^Department of Nephrology, Xi'an No. 4 Hospital, Xi'an, Shaanxi 710004, China

## Abstract

Mono-(2-ethylhexyl) phthalate (MEHP) and genistein are two of the most prevalent endocrine-disrupting chemicals (EDCs) that present in the environment and food. However, how these two EDCs would affect prepubertal Sertoli cells development was rarely studied. In this study, primary prepubertal Sertoli cells were isolated from 22-day-old Sprague Dawley rats and exposed to MEHP at 1 *μ*mol/L, 10 *μ*mol/L, and 100 *μ*mol/L (M1, M10, and M100), genistein at 10 *μ*mol/L (G), and their combination (G + M1, G + M10, and G + M100). Cell proliferation inhibition rate, apoptosis and necrosis rate, and cellular redox state were evaluated. Our results revealed that MEHP could significantly increase cell proliferation inhibition rate, apoptosis rate, necrosis rate, and intracellular reactive oxidative species level. However, coadministration of genistein could partially alleviate MEHP-induced prepubertal Sertoli cells oxidative injuries via enhancement of testicular antioxidative enzymes activities and upregulation of Nrf2 and HO-1, indicating that genistein could partially attenuate MEHP-induced prepubertal Sertoli cells damage through antioxidative action and may have promising future on its curative role for attenuating other EDCs-induced reproductive disorders.

## 1. Introduction

It is widely acknowledged that endocrine-disrupting chemicals (EDCs) may interfere with the body's endocrine system and produce adverse developmental, reproductive, neurological, and immune effects in both humans and wildlife [1]. Multiple studies revealed that exposure to EDCs including bisphenol A and beta-cypermethrin was closely related to male reproductive system impairment [2, 3], particularly if exposure occurred during early development [4].

From the time of conception through adulthood, humans are exposed to countless anthropogenic and naturally occurring EDCs. Among them, genistein and mono (2-ethylhexyl) phthalate (MEHP) are two kinds of the most prevalent EDCs found in food and environment. Genistein and daidzein, two major soy isoflavone glucosides, are present at high concentrations in soybeans and soybean-derived products and are major sources of xenooestrogen exposure in both humans and animals. Previous studies revealed isoflavones could mimic the actions of oestrogens and clinical reports did not find any adverse effects on male reproductive physiology [5]. More recently, the antioxidative role of genistein aroused great concern [6]. It is interesting to note that genistein could enhance fertility by promoting acrosome reaction at lower doses but potentially suppress male fertility via suppressing acrosome reaction at higher doses [7]. Isoflavones including genistein were also reported to modulate activity of antioxidative defence system and alleviate the oxidative stress induced by the other EDCs cadmium, TPA [8, 9].

MEHP is the metabolite of the widely used plasticizer di(2-ethylhexyl) phthalate (DEHP); Culty et al. [10] revealed that DEHP and MEHP exerted their antiandrogen effect by suppressing fetal testosterone biosynthesis via peroxisome proliferator-activated receptors (PPARs) activation; Sobarzo et al. [11] found that MEHP induced oxidative stress in prepubertal Sertoli cells through regulating the level of lipoperoxides, glutathione, and glutathione S-transferases (GST). Epidemiological study also showed that urinary oxidative stress marker malondialdehyde (MDA) concentration was closely associated with levels of several DEHP metabolites in prepubertal children, providing evidence of the association between phthalate exposure and oxidative stress among the early teenagers [12].

Previous studies mainly focused on the reproductive outcome after single EDC exposure; however, the fact that categories of various EDCs are in large amounts in the environment makes it urgent to make it clear how EDCs mixtures would affect reproductive system, especially for those acting via different mechanisms. Our most recent study demonstrated that genistein could normalize DEHP induced neonatal effects through antioxidative action, which also revealed coadministration of the two EDCs did not follow classical dose-response effects and highlighted the importance of assessing impacts across a range of doses and ages [13]. Compared to neonatal testis, prepubertal testis was classically described as quiescent while Sertoli cells were the most active testicular cell population as testis size increase during prepuberty was mainly due to Sertoli cells proliferation [14]. The nursing role of Sertoli cells ensures import and export of nutrients, hormones, and other chemicals into the tubules, and in this sense Sertoli cells should be the frontline exposed to EDCs and how EDCs would affect Sertoli cells would have fundamental implication on spermatogenesis. The effects of combined exposure to genistein and MEHP on prepubertal Sertoli cells still need further investigation.

Oxidative stress is a common pathological process involved in the process of EDCs-induced testicular cell injury, which makes it possible to have oxidative stress monitoring as an informative way to study interactions between numerous toxicants and the reproductive consequences [15]. We hypothesized that low dose of genistein exposure would exert its antioxidative role in prepubertal Sertoli cells, which may partially alleviate the toxic effects induced by different doses of MEHP. Herein we examined the parameters including Sertoli cells proliferation, apoptosis and intracellular reactive oxygen species (ROS) level, expression of genes and proteins related to antioxidation and apoptosis, and enzymes involved in the regulation of testicular redox state, hoping to gain insight into the early cellular and molecular events that may drive long-term changes.

## 2. Materials and Methods

### 2.1. Sertoli Cells Isolation, Culture, and Treatment

22-day-old Sprague Dawley rats were used as donors in order to obtain the prepubertal Sertoli cells. Prior to study initiation, the experimental protocol was reviewed and approved by the Committee on Animal Research and Ethics of Xi'an Jiaotong University (Xi'an, China). 22-day-old specific pathogen-free (SPF) Sprague Dawley rats were obtained after weaning from the Experimental Animal Center of Xi'an Jiaotong University.

A two-step enzymatic digestion protocol was used to obtain testicular single-cell suspensions enriched for Sertoli cells [16]. Rat testes were removed, decapsulated, and rinsed twice in sterile phosphate-buffered saline (PBS). After being minced, the testes fragments were digested using 0.25% trypsin (TransGen Biotech, Beijing, China) at 37°C in a rocking incubator for 30 min, and then Dulbecco's Minimum Essential Medium (DMEM, 4.5 * *g glucose/ml) mixed 1 : 1 with Ham's F12 (DMEM-F12) medium containing 10% fetal bovine serum (FBS), penicillin (100 IU/ml), and streptomycin (100 IU/ml) (TransGen Biotech, Beijing, China) was added to terminate the first-step digestion and centrifuged twice at 1500 rpm. In the second step of digestion, 2 ml 0.05% type V collagenase (Sigma-Aldrich Inc., St. Louis, USA) was added to the isolated tubular fragments and further digested at 37°C in a rocking incubator for 30 min. After that, the isolated cell suspension was filtered through a 200-mesh stainless steel filter and centrifuged twice at 1500 rpm for 5 min. Cells were then resuspended using DMEM-F12 medium containing 10% FBS, penicillin (100 IU/ml), and streptomycin (100 IU/ml). Finally, dispersed cells were seeded on cell culture flask at the density of 1.5 × 10^6^ cells/ml and were incubated under the condition of 37°C and 5% CO_2_. After being cultured for 2 days, Sertoli cells attached to the bottom of flask with tiny dendrites protruding while the germ cells floated in the medium and can be removed after changing the medium.

Further identification and purity of Sertoli cells were assessed by immunocytochemistry (IHC) labeled with anti-follicle-stimulating hormone receptor (anti-FSHR) antibody (Bioss Biology Corporation, Beijing, China). The cells were plated onto poly-L-lysine-coated cover slips for 20 min and 4% paraformaldehyde fixed for 15* * min. After washing with 1x PBS for 3 times, the cells were incubated with 5% bovine serum albumin (BSA) and incubated with FSHR antibody (1 : 200) overnight at 4°C. On the second day, the slides were washed 3 times with 1x PBS and were then incubated with Biotin-Labeled Goat Anti-Rabbit IgG (Boster, Wuhan, China) at 37°C for 1 h. After being incubated with streptavidin-biotin complex (SABC, Boster, Wuhan, China) for 20 mins, the positive cells were visualized using the DAB kit (Boster, Wuhan, China).

MEHP (CAS: 4376-20-9, obtained from Accustandard Inc., Connecticut, USA) and genistein (CAS: 446-72-0, obtained from Sigma-Aldrich Inc., St. Louis, USA) were dissolved in 99.5% pure dimethylsulfoxide (DMSO, CAS: 67-68-5, obtained from Sigma-Aldrich Inc., St. Louis, USA) into stock solutions of 100 mM. After being reseeded for 24 h, Sertoli cells were exposed to vehicle (Control), GEN (10 *μ*mol/L, G), MEHP (1, 10, and 100 *μ*mol/L; M1, M10, M100), or GEN + MEHP (10 *μ*mol/L + 1 *μ*mol/L, 10 *μ*mol/L + 10 *μ*mol/L, and 10 *μ*mol/L + 100 *μ*mol/L; G + M1, G + M10, and G + M100) separately. The range of MEHP doses was based on the No Observable Adverse Effect Level (NOAEL) of MEHP for human testicular cells, ranging between 1 *μ*mol/L and 10 *μ*mol/L [17], and we also included the dose higher than NOAEL. The dose of genistein in this research was based on the serum genistein concentration in the soy formula-fed infants, which ranges from 1 *μ*mol/L to 10 *μ*mol/L [18].

### 2.2. MTT Assay to Detect Cell Proliferation Inhibition Rate

Purified Sertoli cells were trypsinized and reseeded in 96-well culture plates. Following adhesion, cells were exposed to GEN (10 *μ*mol/L) and different concentrations of MEHP (1, 10, and 100 *μ*mol/L), or GEN + MEHP (10 *μ*mol/L + 1 *μ*mol/L, 10 *μ*mol/L + 10 *μ*mol/L, and 10 *μ*mol/L + 100 *μ*mol/L) for 24 or 48 h. The control group was treated with solvent only. After incubation, 20 *μ*l of 5 mg/ml MTT (Sigma-Aldrich Inc., St. Louis, USA) was added to each well and the cells were further incubated for 4 h. After removing the media, 150 *μ*l MTT solvent was added. The absorbance was measured on an automated microplate reader (Thermo Fisher Scientific, USA) at 490 nm after agitating cells on orbital shaker for 10 min, and *A* value was recorded. The cell proliferation inhibition rate was calculated as follows: 1 −* A*(treatment)/*A*(control) × 100%.

### 2.3. Flow Cytometry Assay

Following treatment with chemicals for 48 h, Sertoli cells were trypsinized using 0.25% trypsin containing no EDTA (TransGen Biotech, Beijing, China), harvested at 2000 rpm for 5 min, washed twice with cold PBS, and resuspended in 500 *μ*L binding buffer at the concentration of 6 × 10^5^ cells/ml. Next, cells were stained with 1 *μ*g/ml Annexin V-APC and 1 *μ*g/ml 7-AAD (TransGen Biotech, Beijing, China) in the dark for 10 min and analyzed using a Flow Cytometer System (Beckman Coulter, Miami, USA).

### 2.4. ROS Assay

After being treated with chemicals for 48 h on 24-well plate, Sertoli cells were incubated with carboxy-2′,7′-dichloro-dihydro-fluorescein diacetate (DCFH-DA) (Sigma-Aldrich Inc., St. Louis, USA) probe dissolved in DMEM/F12 medium at the concentration of 10 *μ*M at 37°C for 30 min and then washed by DMEM/F12 medium for 5 min, 3 times. Fluorescence was observed and pictures were taken under the fluorescence microscope (Olympus, Tokyo, Japan) at 488 nm (excitation) and 525 nm (emission) wavelengths. Integral optical density value (IOD) was measured using Image-Pro Plus 5.0 (Media Cybernetics, Bethesda, USA).

All assays were performed in at least three individual experiments, each comprising no less than three replicates.

### 2.5. Evaluation of Medium Redox State

After Sertoli cells were treated with chemicals for 48 h, medium was collected for further redox state analysis. Total antioxidant capacity (T-AOC), superoxide dismutase (SOD), total GSH, glutathione disulfide (GSSG), and thiobarbituric acid reactive substances (TBARS) were evaluated using the clinical chemistry assay kits (Nanjing Jiancheng Bioengineering Institute, China) according to the manufacturer's instruction to monitor testicular redox state.

T-AOC was determined by the ferric reducing/antioxidant power assay and detected at 520 nm using the spectrophotometer, the final concentration was expressed as U/ml.

SOD activity was measured by water soluble tetrazolium salts assay (WST-1), which monitored the inhibition rate of SOD to the process of formazan dye formation from tetrazolium salt mediated by the superoxide anion. The absorbance was scanned at 450 nm using a microplate reader. The final result was expressed as U/ml.

T-GSH and GSSG content were measured using dithionitrobenzoic acid reagent and the absorbance was scanned at 405 nm using microplate reader, GSH content was calculated as T-GSH-2 × GSSG, and the final results were expressed as the ratio of GSH/GSSH.

TBARS was measured and the absorbance was measured with the ultraviolet spectrometer at 532 nm against blanks prepared by distilled water, results were expressed as nmol/ml.

### 2.6. RNA Extraction and Quantitative Real-Time PCR

Sertoli cells RNA was extracted using total RNA extraction kit (Fastagen, China). cDNA was synthesized from isolated RNA using RevertAid™ First-Strand cDNA Synthesis Kit (Thermo Fisher Scientific, USA). Quantitative real-time PCR (qPCR) was performed using the Bio-Rad Real-Time PCR System (IQ5, Bio-Rad). Beta-actin was used as an endogenous control and for normalization of gene targets. The relative gene expression was analyzed using the 2^−ΔΔCt^ algorithm. The primers satisfied the criteria that melting temperature (Tm) was around 60~64°C and GC content was around 35~65%. BLAST alignment was run to ensure the selected primers are unique to the desired target sequence. The genes and primer sequences can be found in Table 1.

### 2.7. Western Blot

Total protein was extracted using a Total Protein Extraction Kit (Solarbio, Beijing, China). Proteins were separated on the 12% sodium dodecyl sulfate-polyacrylamide gel and then transferred to a polyvinylidene fluoride membrane. After blocking with 5% milk in 1×Tween 20-phosphate-buffered saline (PBST), the membranes were incubated with specific primary antibodies against rat Nrf2 (Bioworld, 1 : 200), HO-1 (Abcam, 1 : 500), Caspase-3 (Abcam, 1 : 300) and cleaved Caspase-3 (CST, 1 : 300), and *β*-actin (TransGen, 1 : 10 000) diluted in PBST overnight at 4°C, followed by incubation with anti-rabbit IgG conjugated with HRP (TransGen) at a 1 : 2000 dilution (TransGen) or anti-mouse IgG conjugated with HRP (TransGen) at a 1 : 10 000 dilution (*β*-actin) for 1 h and then with Substrate Chemiluminescence Kit (Millipore, Billerica, MA, USA). Images were captured using the Alpha FluorChem E gel documentation system (ProteinSimple, USA).

### 2.8. Statistical Analysis

Data were expressed as (mean ± SEM) and analyzed using SPSS 15.0 (SPSS Inc., Chicago, IL, USA). Normality and homogeneity of variances were evaluated prior to statistical analysis. Data were analyzed by one-way analysis of variance (ANOVA) and multiple comparisons were done between combined exposure groups and control and single-exposure groups by LSD when equal variances were assumed otherwise followed by Games-Howell. Differences were considered to be statistically significant at the probability level of 5% (*P* < 0.05).

## 3. Results and Discussion

### 3.1. Results

#### 3.1.1. Sertoli Cell Identification

As the germ cells mainly floated in the medium while Sertoli cells adhered to the flask, germ cells were removed after changing the medium after being cultured for 2 days. Sertoli cells showed transparent tiny dendrites protruding under microscope.

**Figure 1:**
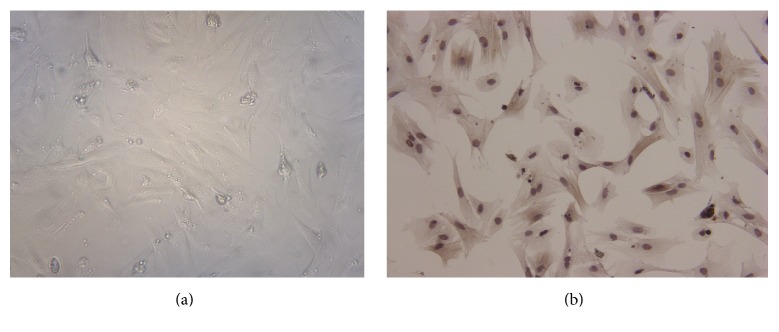
Morphology of Sertoli cells under inverted microscopy (a) and immunohistochemical staining of Sertoli cells with FSHR (b). After changing the medium, Sertoli cells showed tiny dendrites protruding and no germ cells were visible (a). Sertoli cells were positively stained brown with FSHR, and purity of Sertoli cells was >95% (b). 200x magnification. Scale bars indicate 100 *μ*m.

Sertoli cells are mainly activated and regulated by FSH to ensure the normal spermatogenesis in the adult, and this process is mainly mediated by the bonding of FSH and FSH receptor (FSHR), which is uniquely expressed in the membrane of Sertoli cells. Further identification was done by FSHR IHC staining (Figure 1(b)). All primary cells were positively stained brown with FSHR, indicating that they were Sertoli cells and purity of Sertoli cells is >95%. Sertoli cells morphology and IHC staining were shown in Figure 1.

#### 3.1.2. Effects on Cell Proliferation Inhibition Rate

**Figure 2:**
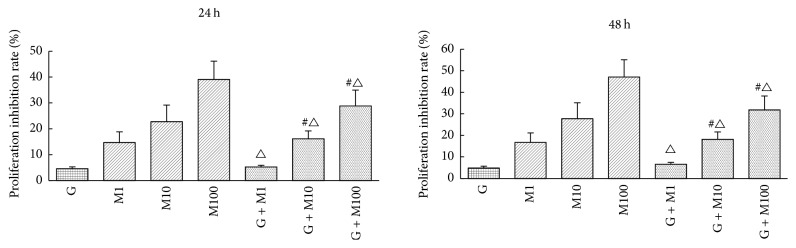
Effects of GEN and MEHP on Sertoli cells proliferation inhibition rate at 24 h and 48 h. ^#^Significantly different from G at *P* < 0.05. ^△^Significantly different from corresponding M at *P* < 0.05.

Cell proliferation inhibition rate at 24 h and 48 h is shown in Figure 2. Coexposure to genistein and MEHP at 1 *μ*mol/L showed significant decrease compared with M1 (*P* < 0.05). Exposure to G + M10 and G + M100 significantly decreased proliferation inhibition rate compared with corresponding MEHP single exposure (*P* < 0.05). Moreover, the calculated half-maximal inhibitory concentration (IC50) of MEHP at 24 and 48 h was 508.90 *μ*M and 154.28 *μ*M, respectively, while the calculated IC50 of MEHP after genistein administration at 24 and 48 h was 826.60 *μ*M and 635.94 *μ*M, respectively, manifesting that though not completely normalized, genistein may alleviate cell proliferative inhibition in prepubertal Sertoli cells development.

#### 3.1.3. Effects on Cell Apoptosis and Necrosis

**Figure 3:**
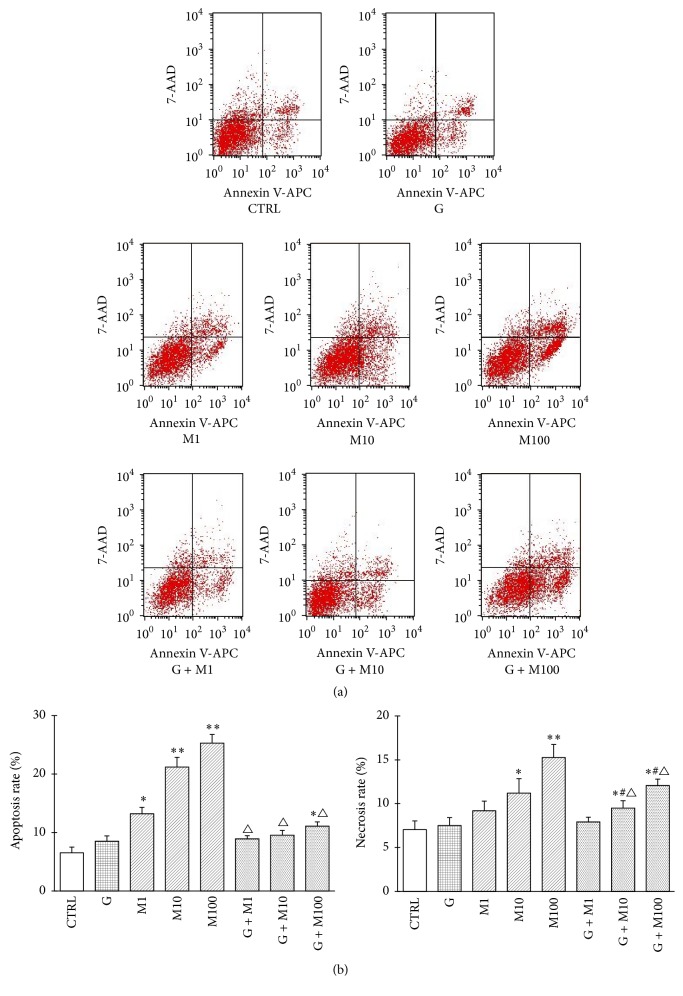
Effects of GEN and MEHP exposure on Sertoli cell apoptosis and necrosis. After being treated for 48 h, Sertoli cells were collected for Annexin V-APC and 7-AAD staining followed by flow cytometry analysis. (a) shows flow cytometric plots. (b) shows flow cytometric analysis result. ^*∗*^Significantly different from control at *P* < 0.05. ^*∗∗*^Significantly different from control at *P* < 0.01. ^#^Significantly different from G at *P* < 0.05. ^△^Significantly different from corresponding M at *P* < 0.05.

Effects of genistein, MEHP, and their combination on Sertoli cells apoptosis and necrosis are shown in Figure 3. The apoptosis rate analysis revealed significant increase in MEHP-treated groups compared with control (*P* < 0.05 or *P* < 0.01). Coexposure to genistein and MEHP caused significant decrease compared with MEHP single exposure (*P* < 0.05), while increased apoptosis rate was still observed in G + M100 compared with control (*P* < 0.05). Similar trend was also found in the necrosis rate, which showed that M10 and M100 significantly increased the necrosis rate compared with control while the combination of G + M10 and G + M100 decreased necrosis rate compared with M10 or M100, manifesting that though not completely normalized, genistein may exert its protective role in prepubertal Sertoli cells development when exposed to MEHP.

#### 3.1.4. Evaluation of ROS Production

**Figure 4:**
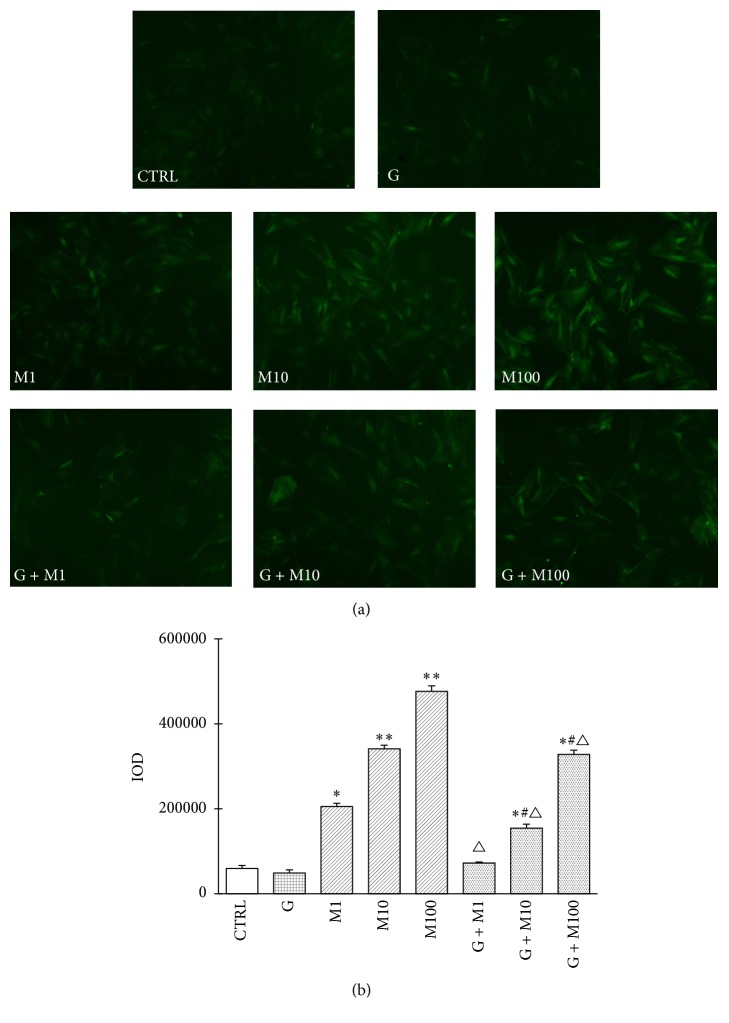
Effects of GEN and MEHP exposure on Sertoli cells ROS production. After being treated for 48 h, Sertoli cells were collected for DCFH-DA staining. (a) shows fluorescence images. (b) shows fluorescence analysis result. IOD = area × average density. ^*∗*^Significantly different from control at *P* < 0.05; ^*∗∗*^significantly different from control at *P* < 0.01; ^#^significantly different from G at *P* < 0.05; and ^△^significantly different from corresponding M at *P* < 0.05.

ROS production after genistein, MEHP, and their combination exposure was shown in Figure 4. The cell permeable DCFH-DA is added to cells and is hydrolyzed by cellular esterases to DCFH. Once oxidized by ROS, DCFH becomes fluorescent DCF. We found that control and genistein exposure showed low ROS production, while exposure to MEHP at 1, 10, and 100 *μ*mol/L caused significant increase of intracellular ROS production compared with control (*P* < 0.05 or *P* < 0.01). The combined exposure of G + M1, G + M10, and G + M100 showed decreased intracellular ROS level compared to corresponding MEHP single exposure (*P* < 0.05); however, significantly higher ROS production was still observed compared with control or genistein group, which was consistent with changes of the proliferation inhibition rate as well as the apoptosis rate, indicating that genistein exerts it protection role by alleviating the ROS production in prepubertal Sertoli cells.

#### 3.1.5. Effects on Medium Redox State

**Figure 5:**
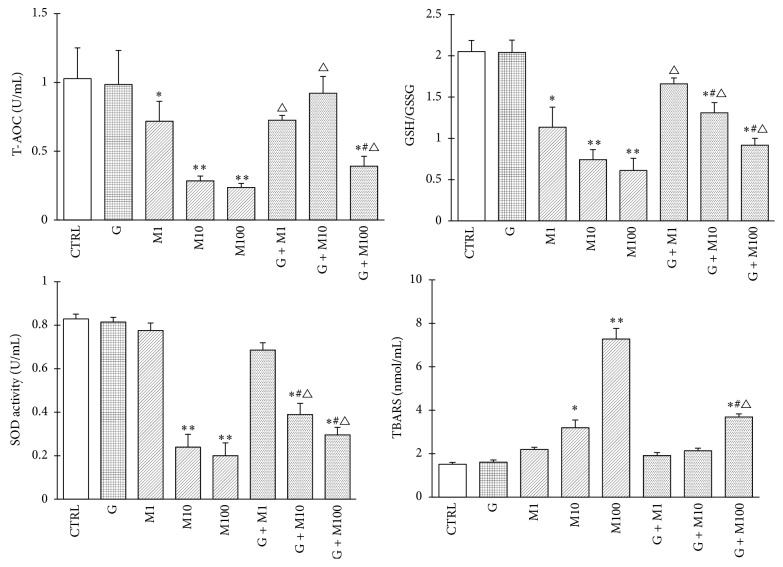
Effects of GEN and MEHP exposure on medium redox state. ^*∗*^Significantly different from control at *P* < 0.05, ^*∗∗*^significantly different from control at *P* < 0.01; ^#^significantly different from G at *P* < 0.05; ^△^significantly different from corresponding M at *P* < 0.05.

Medium redox state in each group at 48 h was shown in Figure 5. MEHP treatment resulted in significant reduction of medium T-AOC, SOD activity, and the ratio of GSH/GSSG, especially at the dose of 10 *μ*m and 100 *μ*M (*P* < 0.05) while the combination of genistein and MEHP exhibited significant increase compared with corresponding single MEHP exposure (*P* < 0.05), showing opposite trends as the changes of intracellular ROS production, which indicates that the antioxidative role of genistein and the enhancement of Sertoli cells antioxidative ability may contribute to the decrease of ROS production. In contrast, TBARS level in M10, and 100 groups increased significantly compared with control (*P* < 0.05) while coadministration of genistein and MEHP showed significant decrease compared with corresponding single MEHP exposure (*P* < 0.05), which may critically depend on the increased antioxidative capacity after coadministration with genistein.

#### 3.1.6. Gene Expression of Nrf2, HO-1, and Caspase-3

**Figure 6:**
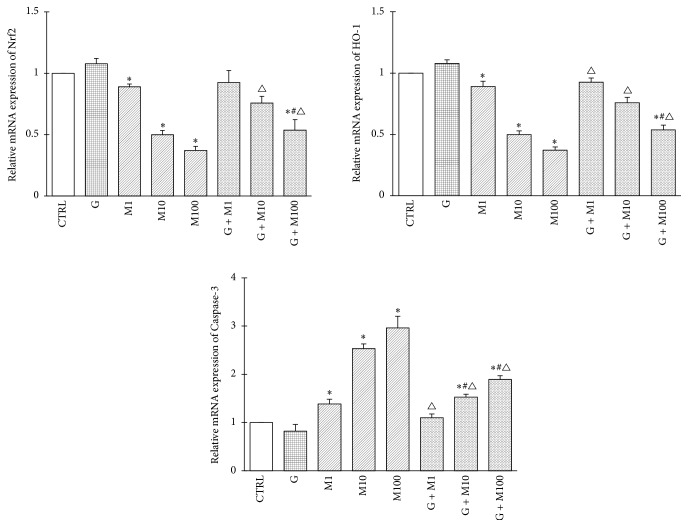
Effects of GEN and MEHP exposure on gene expression of Nrf2, HO-1, and Caspase-3. ^*∗*^Significantly different from control at *P* < 0.05; ^#^significantly different from G at *P* < 0.05; ^△^significantly different from corresponding M at *P* < 0.05.

Gene expression analysis of Nrf2, HO-1, and Caspase-3 of each group is shown in Figure 6. No significant changes were observed between control and Group G. Exposure to MEHP1 and 10 and 100 *μ*M caused significant decrease in Nrf2 and HO-1 expression compared with control (*P* < 0.05), while the combined exposure of genistein and MEHP showed increase in Nrf2 and HO-1 expression compared to corresponding MEHP single exposure (*P* < 0.05), which manifests that although oxidative stress was not completely attenuated, genistein may exert its protective role in prepubertal Sertoli cells via activating Nrf-2 and downstream HO-1. Caspase-3, the marker of cell apoptosis, showed significant increase in M1, M10, and M100 (*P* < 0.05), while the combination of genistein and MEHP decreased significantly compared with MEHP single exposure (*P* < 0.05), although upexpression was still observed compared with control.

#### 3.1.7. Protein Expression of Nrf2, HO-1, Caspase-3, and Cleaved Caspase-3

**Figure 7:**
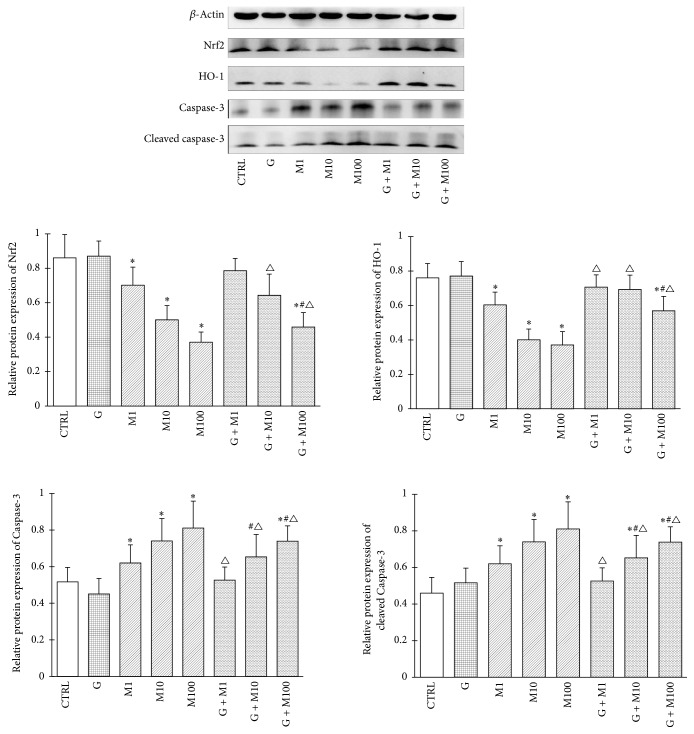
Effects of GEN and MEHP exposure on protein expression of Nrf2, HO-1, Caspase-3, and cleaved Caspase-3. ^*∗*^Significantly different from control at *P* < 0.05; ^#^significantly different from G at *P* < 0.05; and ^△^significantly different from corresponding M at *P* < 0.05.

Alterations of Nrf2, HO-1, and Caspase-3 mRNA were further validated at the protein level by western-blot and subsequent quantitative image analysis (Figure 7). Consistent with gene expression, protein expression of Nrf2 and HO-1 showed similar trends. With the increase of MEHP doses, significant decrease in Nrf2 and HO-1 expression relative to control was observed (*P* < 0.05), while the combination of G + M1, G + M10, and G + M100 showed increase in Nrf2 and HO-1 expression compared to corresponding MEHP single exposure (*P* < 0.05).

Caspase-3 and its activated form of cleaved Caspase-3 were elevated significantly in the three MEHP-treated groups (*P* < 0.05), while the combination with genistein significantly reduced cleaved Caspase-3 expression although upexpression still existed compared with control (*P* < 0.05), which further indicates that genistein could partially attenuate Sertoli cell apoptosis in prepubertal testis, in which process Nrf-2 and downstream HO-1 upregulation may play a vital role in alleviating MEHP-induced oxidative stress.

## 4. Discussion

The prepubertal testis has classically been defined as a quiescent organ, but recent findings revealed that prepuberty is a critical window for male reproductive system development; during this stage the process of testicular spermatogenesis and steroidogenesis is highly responsive to EDCs [19], finally resulting in disturbed spermatogenesis and higher incidence of testicular germ cell cancer [20]. During the fetal and neonatal period, Sertoli cells keep intensively proliferating until postnatal day 15 in rats and then Sertoli cells go into relatively quiescent prepubertal stage [21]. Any disturbance that interferes with Sertoli cells maturation leading to functional impairment and decrease of cell number would detrimentally deteriorate testicular function and the spermatogenic potential in the adult [22]. However, the relative scarcity of studies on this stage and the imminent concern on EDCs mixture exposure make it urgent to investigate the possible acute effects on testis cells development and other reproductive parameters [19].

The present study showed that single exposure to MEHP, especially at high doses, induced elevated prepubertal Sertoli cells proliferation inhibition rate as well as the apoptosis rate, accompanied by increased intracellular oxidative stress and disturbed antioxidative enzymes activities. For peripubertal animals, MEHP was confirmed to disrupt Sertoli cells cytoskeleton and tight junctions followed by increased apoptosis and detachment from germ cells, in which process Sertoli cells number would limit germ cell population [11]. Compared to adult, peripubertal rodents are particularly sensitive to phthalate-induced testicular injury. The mechanism that MEHP-induced testis toxicity includes decreased Sertoli cell number and impaired cell function, which may detrimentally affect the first wave of spermatogenesis starting around prepuberty [23]. In vivo study showed that MEHP administration led to loss of communicating interactions between Sertoli cells and germ cells in the rats [24]. In the in vitro study using rat testis organotypic culture model, MEHP at 10 *μ*M caused decreased Sertoli cell proliferation [25] and MEHP at 100 *μ*M disrupted Sertoli cell maturity in neonatal rat testis cultures [26]. In our in vivo study focusing on prepubertal rats exposed to GEN and DEHP, we also found Sertoli cells were the main target of phthalates toxicity, of which the most significant histological abnormality we found was the tubular vacuolation, indicative of a breakdown in Sertoli-germ cell junctions [27]. Since Sertoli cells are capable of supporting a finite number of germ cells through cell-cell contact and maintaining the integrity of the epithelium, the decreased Sertoli cell number and functional deterioration established during prepuberty will inevitably lead to interruption in the differentiation of germinal cells or even the spermatogenesis arrest. Interestingly, the combined exposure to genistein and MEHP showed significant decrease in cell proliferation inhibition rate, apoptosis rate, and oxidative stress markers compared with MEHP single exposure. It is reasonable to speculate that genistein may exert protective role in its combination with MEHP, which acts in the way different from their classical dose-responsive way, highlighting the importance of assessing impacts across a range of doses, ages, and mixtures.

Redox control of testicular cell physiology is one of the most important regulatory mechanisms. In normal physiological state, potent antioxidant system protects testicular cells against ROS damage, but EDCs exposure could probably cause imbalance in prooxidant/antioxidant content and result in oxidative injuries [28]. DEHP and MEHP have been proven to have deleterious effects on the male reproductive system via inducing dramatic changes in germ cells, Sertoli cells, and Leydig cells [11, 29, 30]. However, the underlying mechanism by which MEHP exerts toxic effects on reproductive system has not yet been fully elucidated. Our results showed that exposure to consecutive three doses of MEHP caused impairment of antioxidative enzyme activities, alternation of the ratio of GSH/GSSG, and the increase of TBARS. The medium antioxidative enzymes activities were closely related to the dose of MEHP, which showed aggravation of oxidative stress as the doses increase. Culty et al. [10] reported peroxisome proliferator-activated receptors (PPARs) activation played a key role in suppressing fetal testosterone biosynthesis after phthalates exposure, but more recently oxidative stress was found to medicate phthalates induced testicular dysfunction, in which process enzymatic and nonenzymatic cellular antioxidants were disturbed accompanied by elevated level of reactive oxygen species production and DNA damage [31]. Kasahara et al. [32] found that DEHP enhanced the generation of ROS and selectively decreased GSH and ascorbic acid in the testis, thereby inducing apoptosis of spermatocytes to cause atrophy of testis. In the study conducted to exam sperm function in adult rats after low-dose exposure to DEHP during adolescence, Hsu et al. [33] found significant increase of hydrogen peroxide (H_2_O_2_) generation in the sperm of the 1000 *μ*g/kg DEHP-treated group, accompanied by higher percentage of sperm with tail abnormality and sperm DNA fragmentation index.

For combined exposures, the mixtures may act not simply in adding or subtracting manners of individual components [15]. The combined exposure of genistein and MEHP showed upregulated antioxidative enzymes activities and downregulated TBARS production compared with DEHP-single exposure. Even though genistein seems to help testis recover form MEHP-induced Sertoli cell injuries, the combined exposure of G + M10 and G + M100, however, still showed downregulated enzymes activities and increase of TBARS level compared to control, suggesting that genistein exposure could partially attenuate MEHP-induced acute alterations in prepubertal Sertoli cells by enhancing cellular antioxidative ability. Genistein is believed to have both estrogenic [5] and antioxidative effects [34]. Recent study revealed that genistein could improve T-AOC while decreasing protein carbonyl and TBARS levels in genistein-treated nephrotic rats [35], and similar findings were also reported in the other in vitro and in vivo studies [36, 37]. Although there were combined studies of genistein with other EDCs in early researches, the study on the combination of MEHP and genistein has been lacking. Our most recent study (collaborating with Martine Culty's group) examined testicular effects after gestational exposure to DEHP at relatively low dose and revealed that the redox markers (Nqo1, Sod2, Sod3, Trx, Gst, and Cat) were significantly altered at PND3, while being attenuated when combined with GEN, suggesting the involvement of cellular stress in short-term DEHP effects and a protective effect of GEN [13].

We further investigated the expression of genes and proteins involved in the process of cell antioxidative defence and apoptosis. The present study showed that single exposure to MEHP, especially at high doses, induced the downregulation of Nrf2 and HO-1 expression while caspase-3 was found to be upregulated. By contrast, cotreatment with genistein attenuated these effects. In the event of oxidative stress, stress-sensing cysteine in cytoplasmic KEAP1 changes the conformation and subsequently dissociates with Nrf2, followed by Nrf2 migration to the nucleus where it heterodimerizes with small Maf protein and binds to ARE, eventually resulting in the transcriptional regulation of target genes [38]. Of all the target genes, HO-1 has been reported to be observed exclusively in the Sertoli cells in rats and play a role in normal spermatogenesis [39]. Different from the other isomers including HO-2 or HO-3, HO-1 is an inducible type of HO and detectable under normal condition. Moreover, HO-1 is believed to have the most AREs on its promoter, making it highly protective by degrading its prooxidant substrate, heme, and enhancing the antioxidants biliverdin and bilirubin production. Our results suggest that genistein functions as an antioxidant in the testes and suppresses apoptosis by acting as scavengers of ROS via activating Nrf2 and HO-1, exerting protection against MEHP-induced Sertoli cell toxicity, which is consistent with the morphological and histological changes in our in vivo study. Moreover, assessing reproductive risk based on single chemical effect might not faithfully represent the true outcome of mixture exposure during critical periods of male reproductive development. Future experiments will involve detailed analysis of cellular and molecular events contributing to acute as well as chronic effects in testis development including epigenetic aberrations that may exert long-term perturbations in gene expression.

## 5. Conclusions

In this study, our results revealed that MEHP could disrupt prepubertal Sertoli cells proliferation by increasing intracellular ROS level especially at high doses. However, coadministration of genistein could partially alleviate MEHP-induced prepubertal Sertoli cells damage via enhancement of testicular antioxidative enzymes activities and upregulation of Nrf2 and HO-1. Cotreatment of genistein devoid of endocrine-disrupting side effects may have promising future on its curative role for attenuating other EDCs-induced reproductive disorders. Based on the results, it can be speculated that dietary intake of isoflavones may make the prepubertal testes less susceptible to phthalates induced injury.

## Figures and Tables

**Table 1:** Primer sets used for quantitative real-time PCR.

Gene name	Accession number	Forward primer	Reverse primer
*β*-Actin	NM_031144.2	5-CTATCGGCAATGAGCGGTTCC-3	5-TGTGTTGGCATAGAGGTCTTTACG-3
Nrf2	NM_031789.2	5-ACGGTGGAGTTCAATGAC-3	5-TGTTGGCTGTGCTTTAGG-3
HO-1	NM_012580.2	5-GAAGAGGAGATAGAGCGAAAC-3	5-TGTGGCTGGTGTGTAAGG-3
Caspase-3	NM_012922.2	5-TGGAACGAACGGACCTGTG-3	5-CGGGTGCGGTAGAGTAAGC-3
